# Supporting Transitions from Hospital to Home by Engaging Volunteers of Third Sector Organizations: A Scoping Review

**DOI:** 10.5334/ijic.9796

**Published:** 2025-12-02

**Authors:** Michelle L. A. Nelson, Alana Armas, Marianne Saragosa, Evan MacEachern, Simrit Jhajj, Rachel Thombs, Shannon Thom, Rambel Palsis, Oya Pakkal, Hardeep Singh, Heather Cunningham

**Affiliations:** 1 Lunenfeld-Tanenbaum Research Institute, Sinai Health System, Toronto, Ontario, Canada; 2 Institute of Health Policy, Management, and Evaluation, Dalla Lana School of Public Health, University of Toronto, Toronto, Ontario, Canada; 3 March of Dimes Canada, Toronto, Ontario, Canada; 4 Department of Occupational Therapy, College of Rehabilitation Sciences, University of Manitoba, Winnipeg, Manitoba, Canada; 5 Gerstein Science Information Centre, University of Toronto, Toronto, Ontario, Canada

**Keywords:** Volunteer, third-sector, transitional care, community, public health, scoping review

## Abstract

**Introduction:**

The transition from hospital to home is a critical clinical juncture marked by significant risks. Third Sector Organizations (TSOs) are well-positioned to support these transitions through volunteer-based programs. Given the increasing complexity of patient needs and the push for reduced hospital lengths of stay, the integration of community resources into transitional care becomes vital.

**Objective:**

Study objectives were i) to identify where TSOs are engaged in supporting post-hospital transitions, ii) to document the characteristics of transitional care models delivered by TSOs, and iii) to characterize the clients participating in these volunteer-supported programs.

**Methods and Results:**

Forty-eight articles that reported on a community-based program delivered by a third-sector organization supporting adults transitioning from hospital to home were included. Study results suggest that TSOs can fill critical gaps in transitional care by leveraging local knowledge and providing personalized, practical, and psychosocial support. TSOs leveraged volunteers to offer personalized, community-based support that addressed both practical and psychosocial needs during care transitions; however, significant variability in program structure and limited evaluation data hindered the assessment of effectiveness and transferability. All programs were time-limited, engaged volunteers in service delivery, and provided home-based and community-based services.

**Conclusions:**

This review highlights the importance of integrating volunteers and TSOs into health systems to develop a more comprehensive approach to transitional care. However, the scalability of volunteer and third-sector-facilitated programs may be challenged by a lack of consistency in programs and reporting, which can undermine transferability and evidence-based practice.

## Introduction

Patients continue to be discharged from hospitals ‘quicker and sicker.’ Avoiding a prolonged hospital stay and its associated risks is essential [1]. However, we must also recognize the transition from hospital to home as a time of vulnerability [2,3,4,5,6,7,8,9]. When the transition from hospital to home is not well managed, people are vulnerable to adverse events, medication errors, hospital readmission, or even death [10,11,12,13,14,15]. These risks disproportionately affect older adults, people with complex conditions, or marginalized populations [16]. Elevated rates of morbidity and mortality among vulnerable populations during the transition from hospital to home underscore the need for support systems to minimize these risks [17,18,19].

Efforts to address issues in care transitions are evident across the health and social care sectors [20,21,22] [18,19], many of which were instigated to compensate for decreasing lengths of stay and increasingly complex patient populations [23]. While many of these transitional care interventions have proven effective [24], they have predominantly focused on a subset of the broader population who require services, are typically delivered by health care providers, and are time-limited interventions offered in select settings [25]. This leaves a large proportion of individuals who may have longer-term needs, live in rural or remote locations, or require culturally tailored interventions underserved. health system leaders are under pressure to develop new ways of meeting the complex needs of patients.

While clinical interventions during care transitions focus primarily on medical management, patients often face practical and social challenges that fall outside the scope of formal healthcare services. Health systems increasingly recognize that addressing these gaps requires leveraging existing community assets and social infrastructure [26,27], yet the mechanisms and contributions of these community partnerships in supporting care transitions remain poorly understood.

Community organizations are often described as agencies that fall within the ‘Third Sector’ – a term used to describe the wide variety of organizations characterized as neither public nor private sectors and includes voluntary and community organizations, such as registered charities and associations, self-help and community groups, and social enterprises [28,29]. Given their global recognition and impact on local communities, the concept of Third Sector Organizations (TSOs) within care transitions warrants further exploration.

Third-sector organizations are situated within local communities and focus on meeting the needs of community members through non-profit-generating activities. They are uniquely positioned by their mandates and operational functions, which may be particularly useful in supporting those transitioning from hospital to home. TSOs also have a deep understanding of their local community, partly by leveraging the knowledge and rapport of local volunteers. Accordingly, community members view TSOs as a trusted entity that is well-positioned to help [30], enabling a connection to ‘hardly reached’ people and a reputation for upholding expected program benefits. TSOs can develop programs that target support and resources to address social determinants of health – issues that may disproportionately impact an individual’s transition from hospital to home compared to peers. With an understanding of the unique needs, preferences, and challenges different communities face, these organizations can offer more personalized and practical support to individuals from marginalized or underserved populations.

A significant function of TSOs’ successful outreach is recruiting community members as volunteers to address health inequities, improve system access and performance [31], and meet population health needs. Indeed, volunteers contribute to specialized knowledge of community needs and reduce human resource costs [32]. For the past decade, influential organizations [33,34], have advocated for more purposeful engagement of TSOs in healthcare delivery. While research on the engagement of community organizations in care transitions is emerging, the extent and nature of their participation still require further investigation [35,36,37]. Therefore, this scoping review synthesized the available literature focused on the contributions of TSOs in supporting care transitions from hospital to home to address the following question:

*How, where, and for which populations have third-sector organizations engaged volunteers in programs to support adults’ transition from hospital to home?*

Study objectives included:

1. To determine where TSOs have been engaged to support adults in the transition home after hospital discharge.
2. To document program characteristics of transitional models delivered by volunteers of the TSOs.
3. To identify the characteristics of clients participating in community-based volunteer-supported transition programs.

A scoping review methodology was selected rather than a systematic review because the objective was to map the breadth and nature of TSO involvement in transitional care across diverse contexts and populations, rather than to assess the effectiveness of specific interventions. Additionally, the heterogeneous nature of volunteer-supported programs precluded meaningful quantitative synthesis.

## Methods

The methods for this scoping review were previously published [38] and are briefly described below. This scoping review follows the PRISMA (Preferred Reporting Items for Systematic Reviews and Meta-Analyses) extension for scoping review guidelines [39]. Ethics Review Board approval was not required. Individuals with lived experience were not involved in the conduct of the knowledge synthesis, however individuals with expertise by experience (embedded scholars, program managers and volunteers) were included on the research team.

### Search Strategy and Information Sources

**Figure 1:**
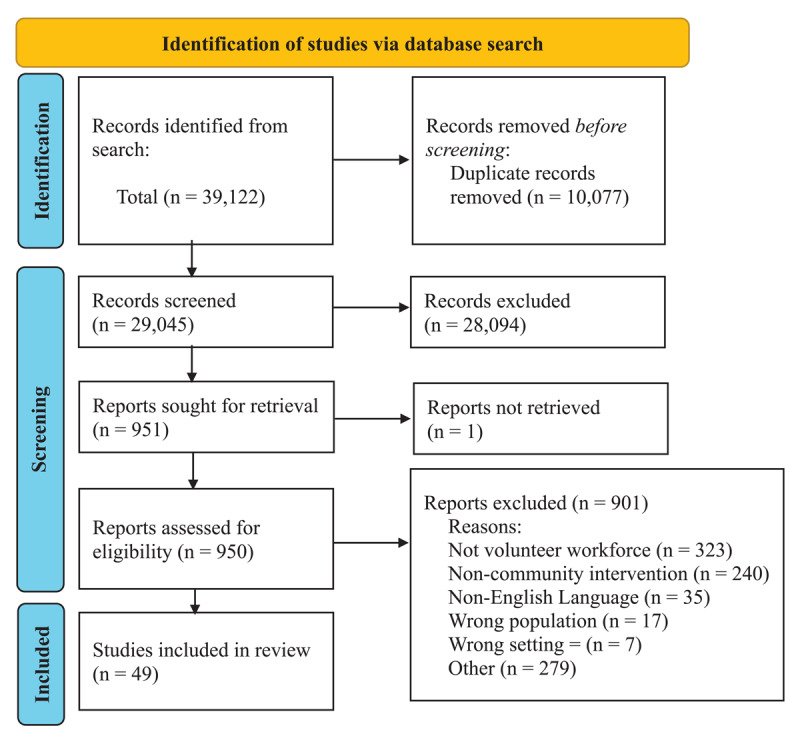
PRISMA flowchart of article screening.

In collaboration with an Information Scientist (HC), a comprehensive search strategy was initiated using MeSH terms across the following databases: OVID Medline, EMBASE, PsycInfo, Joanna Briggs Institute (JBI), Social Work Abstracts, Sociological Abstracts, CINAHL, Cochrane Reviews, Ageline, and Scopus. The initial searches were completed in 2019 and updated annually until December 2023. The grey literature search comprised theses and dissertations (ProQuest), relevant newspaper articles, and targeted Google searches, with filters adjusted to exclude sites outside the included high-income countries. To search relevant organizations’ websites, the research team used the grey literature approach from the Canadian Agency for Drugs and Technologies in Health [40], which includes an extensive list of national and international organizations. The research team reviewed and refined the list of organizations. Finally, the team searched the reference lists of relevant reviews in the electronic database searches and consulted experts in transitional care to identify all relevant literature. Studies were compiled and stored using the reference management software package EndNote, and duplicate citations were removed. Figure 1 contains the PRISMA flowchart.

### Eligibility Criteria

*Population*: We considered any program that supports adults (age > 18) transitioning from the hospital to home. As this review focused on the program characteristics of transitional support interventions, it included all volunteer-supported hospital-to-home transition programs available to any adult population to ensure that the search strategy retrieves the most comprehensive pool of relevant literature. However, if an article did not explicitly state the population but met all other inclusion criteria, we included it to capture all relevant literature.

*Concept*: Volunteer-supported transition from hospital to home. We included interventions that used volunteers focused on this transition. For this review, volunteers were defined as individuals undertaking activities or providing services to others in a formal role with a TSO without remuneration for their time or talent [41].

*Context*: Any program provided within a ‘third sector’ organization was eligible for inclusion. The third sector describes organizations that are non-government, nonprofit, charitable, faith-based, or social enterprises.

*Types of evidence to be included*: A date range from 2000 to 2023 was determined as appropriate, to ensure we have sufficient citations, and to summarize program characteristics relevant to current-day health service delivery. Published and unpublished literature, including research from any study design, educational materials, and reports. Grey literature also included unpublished research, program summaries, evaluation reports, theses, organizational reports, and conference proceedings. Literature was limited to only those published in English.

### Study Selection

The citations were screened using a 2-step process. The inclusion and exclusion criteria were pilot-tested on 100 randomly selected articles, after which members of the research team (AA, OP, ST, SJ) reviewed two sets of fifty randomly selected articles at which point a Kappa coefficient of 0.77 was reached. An inter-rater reliability of 0.77 is considered a substantial level of agreement [42]. The titles and abstracts were screened in duplicate by the aforementioned independent reviewers, followed by full-text screening conducted in duplicate. Independent reviewers used Covidence as a reference manager to keep track of their decisions. Discrepancies were resolved by discussion between reviewers and adjudicated by the lead author, MN.

### Data Abstraction

Four research team members (AA, OP, ST, SJ) obtained, reviewed, and charted a copy of each article/document using a pilot-tested extraction form before use. A senior research team member (MN) reviewed and resolved disagreements. Table S1 in the supplementary materials outlines the data abstracted from each article.

### Analysis

Data analysis consisted of a descriptive analysis focused on study and program characteristics undertaken through a qualitative content analysis where findings, such as study characteristics, reported findings, program characteristics, and outcomes, were deductively categorized using the extraction template [43]. The qualitative content analysis was conducted primarily by AA and reviewed and verified with other research team members throughout the analysis.

Preliminary results were presented and discussed at an international conference and with a TSO. Based on the observations, additional questions, and reflections from these discussions, the research team further refined the descriptive findings and content analysis to understand the potential relationship between specific characteristics, such as delivery mechanism, program location, and client characteristics.

## Results

Our literature search yielded 39,122 articles; after de-duplicates (n = 10,077), 29,045 articles were screened. After level one screening, 950 articles were included for full-text screening. Of these 950 articles, 901 were excluded for reasons including no mention of volunteer workforce (n = 323), not being a community-based intervention (n = 240), being published in a language other than English (n = 35), including a population different from the one focused on in this review (n = 17), or was the wrong setting (n = 7). After level two screening, data abstraction was completed for 49 articles. The PRISMA flowchart is shown in Figure 1.

## Article Characteristics

**Figure 2:**
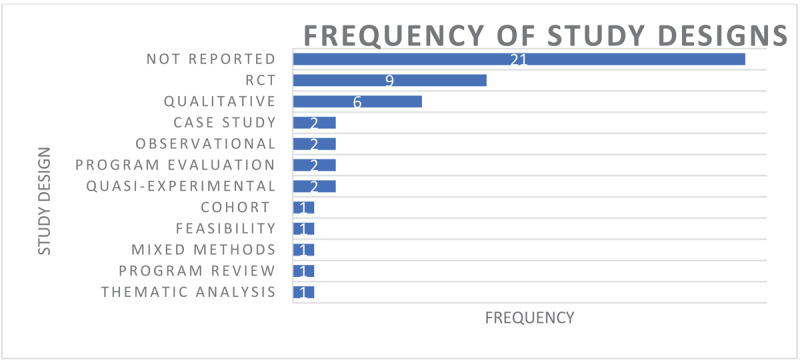
Frequency of study designs from included articles.

Of the 49 included articles, 34 (69.3%) were retrieved from academic databases, and 15 (30.6%) were retrieved from the grey literature search. The most common publication types were original research papers (n = 26) [30,44,45,46,47,48,49,50,51,52,53,54,55,56,57,58,59,60,61,62,63,64,65,66,67,68,69] and news articles (n = 13) [70,71,72,73,74,75,76,77,78,79,80,81,82]. Articles were published between 2000 and 2023, with the number of publications per year trending upward until around 2016, when it plateaued at 2–3 publications per year. Study designs were varied; the most commonly reported study designs were randomized controlled trials (RCT) (n = 9) [44,46,52,57,58,61,62,63,65] followed by qualitative designs (n = 6; Figure 2) [48,49,55,56,66,83].

Study results are presented by study objectives:

**Objective One:**

1. To determine **where** TSOs have been engaged to support adults in the transition home after hospital discharge.

Included articles represented seven geographic locations: the United Kingdom (n = 26) [45,46,47,54,55,56,58,60,61,64,66,71,75,76,77,78,79,80], the United States (n = 10) [48,49,59,63,65,67,70,84,85,86], Canada (n = 7) [50,57,68,69,72,83,87], Finland (n = 1) [44], and Hong Kong (n = 4) [51,52,53,62]. Two articles reported on multiple international study sites, representing Australia and the UK [88] and the USA and Canada [89], respectively. The majority (n = 34) of articles were academic literature (n = 26; Table 1).

**Table 1:** Location and publication classification of included articles.


PROGRAM COUNTRY LOCATION	CITATION	

Australia & UK	Battersby [88]	

United States	Buys, Campbell [63], Crane-Okada, Freeman [65], Dye, Willoughby [67], Hintgen, Radichel [48], Hung, Truong [49], Strait, Fitzgerald [59], Anonymous [70], Bjorklund [84], DeForge and Belcher [85], Schimpf [86]	

Canada	Eaton, Carusone [68], Eaton, Chan Carusone [69], Kessler, Egan [50], Parry, Watt-Watson [57], Dobson [72], Anderson [83], Forchuk, Solomon [87]	

USA & Canada	Badger, Acton [89]	

Finland	Anttila, Huhtala [44]	

Hong Kong	Lai, Wong [51], Law, Lok [52], Lou, Cheng [53], Wong, Ho [62]	

United Kingdom	Brookfield and Mead [55], Cooper, Jehu [64], Dugmore [66], Elston, Gradinger [45], Gillard, Bremner [46], Gradinger, Elston [47], Mazumdar, Ciravegna [54], McLeod, Bywaters [56], Reynolds, Lauder [58], Waddington and Henwood [60], White, Bhattacharya [61], Bubb [71], Cottam [76], Anonymous [75], Anonymous [77], Anonymous [78], Anonymous [79], Anonymous [80], Anonymous [81], Byrne [82], Wright [73], O’Grady [74], Adams [90], Dickinson and Neal [91], Rivers [92]	

**PUBLICATION CLASSIFICATION**	**PUBLICATION TYPE**	**NUMBER**

Academic literature (n = 34)	Research paper	26 [30,44,45,46,47,48,49,50,51,52,53,54,55,56,57,58,59,60,61,62,63,64,65,66,67,68,69]

	Discussion paper	2 [85,89]

	Expert commentary	1 [88]

	Program description	3 [30,86,87]

	Short form report	1 [84]

	Thesis	1 [83]

Grey literature (n = 15)	News article	13 [70,71,72,73,74,75,76,77,78,79,80,81,82]

	Practice Guide	1 [81]

	Organization report	1 [90]


**Objective Two:** Document **program characteristics** of transitional models delivered by volunteers of the TSOs.

### Program Delivery Model

Thirty-seven articles reported having an in-person component to delivering the program, with 28 programs delivered in person [44,46,51,53,55,56,58,60,61,63,66,69,70,72,74,75,77,79,80,82,84,85,86,87,88,92]. Programs also used mixed delivery methods, such as in person and by telephone (n = 10) [48,49,52,57,59,62,67,69,78,91], and in person and virtual (n = 3) [54,64,89], while two articles reported on programs delivered exclusively by telephone [50,65].

Volunteer programs were most often delivered one-to-one with participants (n = 37) [46,47,48,49,50,51,52,53,54,56,57,58,59,60,61,62,63,64,65,67,68,69,70,73,74,75,77,78,79,80,82,85,86,91,92]. Two programs provided individual and group services [84,89], and four were delivered in a group setting [55,66,87,88] (Table 2). Most volunteer programs were designated as time-limited services, although significant variability was in the duration, frequency of contact and service hours provided. Twenty-four programs provided details [45,46,47,49,50,51,52,54,56,57,58,60,61,62,63,64,65,67,69,81,88,91,92], while noting that the programs may adapt service duration to clients’ needs, stating that the length of time varied or had a set program length but offered ongoing support as needed. The remaining articles were categorized as either unclear [44,53,55,68,76,87], not applicable [66,83,85,89], or did not provide information on program duration [48,59,70,71,84,86,90]. Some of these programs adapted service duration. There was notable variation in program duration, ranging from 10 days [63] to 17 months [45,51]. Eighteen articles discussed the frequency of their program visits, with significant variation among them. Four programs provided daily visits or interactions [63,74,81,86], nine reported weekly visits [46,50,52,56,61,62,67,68,69], two reported bi-weekly visits [64,66], two reported monthly visits [51,59], and the remaining article reported visitation “as needed” [49].

**Table 2:** Program characteristics.


SERVICE TYPE	CITATION

Emotional support and social interactions	Dickinson, Allen [30], Anttila, Huhtala [44], Brookfield and Mead [55], Cooper, Jehu [64], Crane-Okada, Freeman [65], Dugmore [66], Gillard, Bremner [46], Hintgen, Radichel [48], Kessler, Egan [50], Law, Lok [52], Lou, Cheng [53], Reynolds, Lauder [58], Waddington and Henwood [60], White, Bhattacharya [61], Anonymous [70], Bubb [71], Anonymous [75], Anonymous [77], Anonymous [78], Anonymous [79], Anonymous [80], Anonymous [81], Byrne [82], Dobson [72], Rivers [92], Bjorklund [84], DeForge and Belcher [85], Forchuk, Solomon [87], Badger, Acton [89]

Completing tasks outside the home	Hung, Truong [49], Reynolds, Lauder [58], Anonymous [70], Cottam [76], Anonymous [75], Anonymous [78], Anonymous [79], Anonymous [80], Byrne [82], O’Grady [74], Dickinson and Neal [91], Rivers [92]

Completing tasks inside the home	Buys, Campbell [63], Hintgen, Radichel [48], Hung, Truong [49], Mazumdar, Ciravegna [54], Reynolds, Lauder [58], Anonymous [70], Cottam [76], Anonymous [78], Anonymous [80], Dobson [72], O’Grady [74], Adams [90], Schimpf [86]

Health care services	Buys, Campbell [63], Cooper, Jehu [64], Dugmore [66], Dye, Willoughby [67], Eaton, Chan Carusone [69], Kessler, Egan [50], Mazumdar, Ciravegna [54], Parry, Watt-Watson [57], Strait, Fitzgerald [59], Wong, Ho [62], Anonymous [70], Byrne [82], Battersby [88], Badger, Acton [89]

Caregiver respite	Dickinson, Allen [30], Anonymous [79], Wright [73]

**PROGRAM DURATION**	

10 days	Buys, Campbell [63]

4 weeks	Wong, Ho [62]

6 weeks	Eaton, Chan Carusone [69], Waddington and Henwood [60], Rivers [92], Battersby [88]

8 weeks	Law, Lok [52], McLeod, Bywaters [56], Parry, Watt-Watson [57], Wright [73]

10 weeks	Crane-Okada, Freeman [65]

12 weeks	Cooper, Jehu [64], Gradinger, Elston [47]

4 months	Dye, Willoughby [67], Gillard, Bremner [46], White, Bhattacharya [61]

5 months	Reynolds, Lauder [58], Dickinson and Neal [91]

6 months	Hung, Truong [49]

12 months	Kessler, Egan [50], Mazumdar, Ciravegna [54], Anonymous [81]

17 months	Elston, Gradinger [45], Lai, Wong [51]

Unclear	Anttila, Huhtala [44], Brookfield and Mead [55], Eaton, Carusone [68], Lou, Cheng [53], Cottam [76], Anonymous [75], Anonymous [77], Anonymous [78], Forchuk, Solomon [87]

Not applicable	Dugmore [66], Dobson [72], Anderson [83], DeForge and Belcher [85], Badger, Acton [89]

Information not provided	Hintgen, Radichel [48], Strait, Fitzgerald [59], Anonymous [70], Bubb [71], Anonymous [78], Anonymous [79], Anonymous [80], Byrne [82], O’Grady [74], Adams [90], Bjorklund [84], Schimpf [86]

**KEY SERVICE SUPPORTS**	

Support transitions home	Eaton, Carusone [68], Eaton, Chan Carusone [69], Reynolds, Lauder [58], Wong, Ho [62], Anonymous [70], Anonymous [79], Anonymous [81], Byrne [82], Wright [73], O’Grady [74], DeForge and Belcher [85], Forchuk, Solomon [87]

Provide emotional support/companionship	Crane-Okada, Freeman [65], Kessler, Egan [50], Law, Lok [52], Anonymous [77], Anonymous [78], Bjorklund [84], Badger, Acton [89]

Support social engagement	Brookfield and Mead [55], McLeod, Bywaters [56], Cottam [76]

Develop self-management skills	Cooper, Jehu [64], Dye, Willoughby [67], Dickinson and Neal [91], Battersby [88]

Help regain confidence and independence	Bubb [71], Anonymous [75], Anonymous [77], Anonymous [78]

Support care coordination	Dugmore [66], Elston, Gradinger [45], Gradinger, Elston [47], Hintgen, Radichel [48], Mazumdar, Ciravegna [54], Parry, Watt-Watson [57], Waddington and Henwood [60]

Provide meals	Buys, Campbell [63], Dobson [72], Schimpf [86]

Reduce hospital readmissions	Anttila, Huhtala [44], Gillard, Bremner [46], Hung, Truong [49], Reynolds, Lauder [58], Strait, Fitzgerald [59], White, Bhattacharya [61], Rivers [92]

Home safety	Adams [90]

Spirituality	Lou, Cheng [53]

Information not provided	Lai, Wong [51], Anderson [83]

**SERVICE DELIVERY**	

Individual	Dickinson, Allen [30], Buys, Campbell [63], Cooper, Jehu [64], Crane-Okada, Freeman [65], Dye, Willoughby [67], Eaton, Carusone [68], Eaton, Chan Carusone [69], Gillard, Bremner [46], Gradinger, Elston [47], Hintgen, Radichel [48], Hung, Truong [49], Kessler, Egan [50], Law, Lok [52], Lou, Cheng [53], Mazumdar, Ciravegna [54], McLeod, Bywaters [56], Parry, Watt-Watson [57], Reynolds, Lauder [58], Strait, Fitzgerald [59], Waddington and Henwood [60], White, Bhattacharya [61], Wong, Ho [62], Anonymous [70], Anonymous [75], Anonymous [77], Anonymous [78], Anonymous [79], Anonymous [80], Byrne [82], Dobson [72], Wright [73], O’Grady [74], Rivers [92], DeForge and Belcher [85], Schimpf [86]

Group	Brookfield and Mead [55], Dugmore [66], Forchuk, Solomon [87], Battersby [88]

Individual, Group	Bjorklund [84], Badger, Acton [89]

Info not provided or able to determine	Anttila, Huhtala [44], Elston, Gradinger [45], Bubb [71], Cottam [76], Anonymous [81], Anderson [83], Adams [90]

**WORKFORCE (INCLUDING VOLUNTEERS)**	

Volunteers only	Cooper, Jehu [64], Crane-Okada, Freeman [65], Gillard, Bremner [46], Hintgen, Radichel [48], Lai, Wong [51], Law, Lok [52], Lou, Cheng [53], McLeod, Bywaters [56], Parry, Watt-Watson [57], Waddington and Henwood [60], White, Bhattacharya [61], Anonymous [75], Anonymous [77], Anonymous [78], Anonymous [80], Byrne [82], Dobson [72], Wright [73], O’Grady [74], Rivers [92], Bjorklund [84], Forchuk, Solomon [87]

Allied health professionals	Brookfield and Mead [55], Dugmore [66], Mazumdar, Ciravegna [54], Reynolds, Lauder [58], Wong, Ho [62], Anonymous [70], Badger, Acton [89]

Multidisciplinary teams	Eaton, Carusone [68], Eaton, Chan Carusone [69], Elston, Gradinger [45], Gradinger, Elston [47], Hung, Truong [49], Kessler, Egan [50], Dickinson and Neal [91], DeForge and Belcher [85]

Nurse	Anttila, Huhtala [44], Dye, Willoughby [67], Strait, Fitzgerald [59]

Primary care providers	Buys, Campbell [63]

Paid staff	Cottam [76]

Handyperson	Adams [90]

Information not provided	Anonymous [75], Anonymous [78], Anonymous [81], Anderson [83], Battersby [88]


### Program Activities and Aims

Community programs offered a wide range of support both outside and inside the home. Community-based activities included transportation, errand running, shopping, banking [48,49,58,76,91,92], and signposting individuals to community programs of interest [91], with notable uptake of community transport services [70,91,92]. Home-based services focused on cleaning, cooking or providing meals, pet care, home repairs, and safety modifications [49,63,70,72,90,91]. We observed a trend prioritizing meal support, delivered either through ‘meals-on-wheels’ style programs [72] or volunteers assisting with cooking in residences.

Health and recovery services encompassed care coordination [45,47,48,54,57,60,66], health monitoring [57,66], patient education [50,64,66], and goal-setting [63,88]. These programs often functioned as extensions of health monitoring from nurses or clinical staff. Emotional and social support included companionship, peer matching based on shared interests, social outings, and informal social activities [44,46,48,50,52,53,55,58,61,64,65,66,70,71,75,77,78,81,82,84,85,87,89,91,92]. Three programs specifically provided caregiver respite [73,79,91]. Some articles provided limited detail about the nature of emotional support services [60,92].

Program aims varied considerably. Twelve programs identified hospital-to-home care as their primary focus [58,62,68,69,70,73,74,79,81,82,85,87], while others targeted specific outcomes such as reducing hospital readmissions (n = 7), developing self-management skills (n = 4) [64,67,88,91], or providing emotional support and companionship (n = 7) [50,52,65,77,78,84,89]. Twenty-nine programs offered multiple services across these categories [44,46,48,50,52,53,55,58,60,61,64,65,66,70,71,81,84,85,87,89,91,92].

#### Program Outcomes

In many articles, program outcomes were not available, but where present, we determined that eleven articles aimed to reduce hospital readmissions (n = 11), and six of those programs reported to have reduced hospital readmissions [49,58,62,66,70,80]. Other studies measured healthcare service utilization [70], health care costs [44,70], mortality [70], program retention [64,84], mental health status [47], and well-being [47,58]. In the evaluative data, clients reported independence [75], feeling supported [49], and an overall positive experience [63], which helped to increase their quality of life and ability to make more connections [49]. Clients also reported feeling heard and understood by volunteers through the facilitation of social support offered by the intervention [57].

### Workforce Characteristics and Training

Program teams varied considerably in composition and structure (outlined within Table 2). Twenty-two programs were delivered solely by volunteers [46,48,51,52,53,56,57,60,61,64,65,72,73,74,77,78,80,82,84,86,87,92], while others included allied health professionals [54,58,70] or multidisciplinary teams [49,50,68,80]. Across 18 articles that reported workforce numbers, the total community-based workforce comprised 1, 194 individuals, including 522 volunteers from volunteer-only programs.

Nine articles described services provided by peers with lived experience [50,55,57,64,65,84,85,87,89], who provided information, answered questions, or shared personal experiences to support participants. Some programs matched individuals based on shared interests to strengthen rapport [84] or offered informal social activities like coffee outings [87] or drop-in sessions [66].

Seventeen articles reported volunteer training that ranged from single-day sessions to 8-week programs [46,48,51,52,53,57,61,64,65,87,92]. Training typically covered community resources, trust-building, home safety, and coping strategies. Mental health programs included specialized training on suicide prevention, risk screening, and empathetic communication [52].

**Objective Three:** Identify the **characteristics of clients** participating in community-based volunteer-supported transition programs.

#### Characteristics of Population Served

Older adults (range: 50–96) [55,76] were the most common recipients of the transitions-focused programs and services [44,49,51,53,56,62,63,65,66,70,74,76,77,92], followed by those receiving support for mental health [46,52,58,61,66,81,83,84,85,87] and those with chronic health conditions [50,51,55,64,67,68,69]. See Table 3 for more detail.

**Table 3:** Population Served.


POPULATION SERVED	

Breast cancer	Crane-Okada, Freeman [65]

Burn survivors	Badger, Acton [89]

Carers	Anonymous [79], Anonymous [80]

Chronic conditions	Brookfield and Mead [55], Cooper, Jehu [64], Dye, Willoughby [67], Eaton, Chan Carusone [69], Elston, Gradinger [45], Kessler, Egan [50], Lai, Wong [51]

Low-income communities	Anonymous [70]

Mental health	Dugmore [66], Gillard, Bremner [46], Law, Lok [52], Reynolds, Lauder [58], White, Bhattacharya [61], Anonymous [81], Anderson [83], Bjorklund [84], DeForge and Belcher [85], Forchuk, Solomon [87]

Older adults	Anttila, Huhtala [44], Buys, Campbell [63], Crane-Okada, Freeman [65], Dugmore [66], Hung, Truong [49], Lai, Wong [51], Lou, Cheng [53], McLeod, Bywaters [56], Wong, Ho [62], Cottam [76], Anonymous [77], Anonymous [78], O’Grady [74], Rivers [92]

Vulnerable populations	Byrne [82]

No specific patient population	Elston, Gradinger [45], Gradinger, Elston [47], Hintgen, Radichel [48], Mazumdar, Ciravegna [54], Parry, Watt-Watson [57], Strait, Fitzgerald [59], Waddington and Henwood [60], Bubb [71], Anonymous [75], Dobson [72], Wright [73], Adams [90], Dickinson and Neal [91], Schimpf [86], Battersby [88]


## Discussion

The term ‘third sector’ is used to describe organizations that belong neither to the public (e.g., the state) nor private (e.g., for-profit enterprises) sectors and was coined to describe United Kingdom and North American organizations and social enterprises as a sector, distinct from public and private sectors [93]. While these organizations share common characteristics, the engagement of TSOs and the services they provide within their communities highly depend on the broader socio-political environment in which they are situated [94]. Therefore, existing literature must be reviewed within the socio-political context in which it was generated. Our search yielded 49 articles and 34 academic reports from six jurisdictions. Most were from the UK. Historically, TSOs from the UK have acted as alternative public service providers commissioned by governments to offer various health services [95]. This may explain the relatively high number of UK-based TSO transitional care literature identified in this review, which may limit the transferability of results. Nevertheless, our scoping review draws upon several key findings.

First, hospital-to-home programs substantively engaged a volunteer workforce to support people in their homes and communities, addressing commonly experienced issues during transitions. Most volunteer-supported programs offered psychosocial and in-person practical assistance, including transportation and help with household tasks and shopping activities. The programs described in our review also addressed emotional support and care coordination of transitions from hospital to home, consistent with other literature on volunteer-supported transitions [95]. Furthermore, our review highlights the potential of TSOs to meet the needs of diverse populations despite the limitations in scale and spread associated with localized interventions. The community-based programs were situated for the citizens they served. These programs often draw their paid and unpaid workforce from the community, strengthening the capacity for culturally mindful and safe treatment plans [96]. While there is no ‘one-size-fits-all’ approach to transition from hospital care to home, volunteers are knowledgeable community members who can alleviate some of the noted challenges experienced by patients and family members in navigating these care transitions.

Second, while all articles included volunteers in the program delivery, 23 programs and services (47%) were provided solely by volunteers, and nine articles described services provided by peers. Seventeen articles reported volunteer training specific to the roles fulfilled. This purposeful engagement of volunteers as a programmatic workforce aligns with the World Health Organization’s definition of health workers as “all people engaged in actions whose primary intent is to enhance health,” which encompasses volunteers in this definition [97]. The results of this review highlight the use of patient-facing volunteerism within healthcare interventions and challenge the prevailing belief that volunteers cannot be placed in patient-facing roles, primarily due to concerns about risk, liability, and confidentiality. However, no articles in our review identified a risk, liability, or confidentiality breach in their volunteer-assisted hospital-to-home transition interventions. When we consider the widespread use of thorough vetting processes, as well as comprehensive training and investment in selecting high-quality volunteers, we recognize a health human resource that should not be overlooked.

Thirdly, the reviewed programs served widely heterogeneous populations. Despite the variability in the patient population, the reviewed programs were still highly focused on the individual receiving care; caregivers were explicitly focused on in only three of the included articles. As caregivers are often cited as essential partners in the care transition process, their engagement is fundamental to ensuring high-quality hospital-to-home transitions. Caregivers serve as a valuable source of information retention, mitigating potential threats to patients’ safety and supporting the quality of care. While many care transition interventions have been designed to address medication and symptom management, care coordination, and self-management, few interventions have focused on caregiver needs or goals. Given the increasing reliance on informal caregivers to assist in recovery after discharge [98], Caregiver support in transitional care is an area that warrants further research.

Finally, the reviewed programs reported variability in structure and offerings (i.e., duration, frequency, and services) and included limited program evaluative data. The lack of comprehensive evaluative data and the significant variability in program structure and offerings present a challenge to understanding the effectiveness and transferability of the programs under review. With robust evaluation data, it becomes easier to ascertain the impact and success of the program. Evaluative data is crucial for determining the outcomes of a program, identifying areas for improvement, and making informed decisions about its continuation or expansion. Moreover, the wide range of variations in program structures and offerings, including differences in duration, frequency, and services provided, poses a challenge to the program’s generalizability and transferability. This variability makes it difficult to determine which specific program elements were most effective, for whom, and under what circumstances. Without a clear understanding of the key components that contribute to the program’s success, the ability to replicate or adapt the program to different health conditions, client demographics, geographic locations, or organizational settings is uncertain. Addressing these issues through rigorous evaluation and delineating key program components would provide a deeper understanding of the program’s impact and potential for successful implementation in various settings.

This review includes a comprehensive search strategy across academic and grey literature, dual screening with inter-rater reliability testing, and being the first systematic examination of TSO contributions to hospital-to-home transitions. Key limitations include restriction to English-language publications, significant heterogeneity in program characteristics and reporting quality that limited conclusions about effectiveness, and exclusion of individuals with lived experience from the review process. The variability in program structure and evaluation approaches across studies prevented identification of best practices for volunteer-supported transitional care programs.

While this review demonstrates the potential contributions of volunteer-supported transitional care programs, several implementation challenges warrant consideration. Organizations must invest substantially in volunteer recruitment, screening, training, and ongoing supervision to ensure program quality and safety, yet face inherent challenges related to volunteer availability, turnover, and consistency of service delivery. Furthermore, the reliance on unpaid labour to address gaps in essential health services raises ethical questions about whether volunteer programs mask chronic underfunding of transitional care rather than providing sustainable solutions. These organizational, safety, and equity considerations suggest that while TSO volunteer programs show promise, their integration into health systems requires careful planning, adequate resource allocation, and ongoing evaluation to ensure both effectiveness and sustainability.

## Additional File

The additional file for this article can be found as follows:

10.5334/ijic.9796.s1 Supplementary File. Tables S1–S2 and Appendix.
